# A Low-Dimensional *X*–*ρ*–*A* Allocation Model for Predicting Light-Dependent Biomass, Protein, and Lipid Dynamics in TAP Batch Cultures of *Chlamydomonas reinhardtii* CC-400

**DOI:** 10.3390/molecules31173080

**Published:** 2026-09-01

**Authors:** Lanbo Yi, Dangkun Du, Bingyan Yao, Chunxiu Lin, Bin Liu

**Affiliations:** 1 School of Mechanics and Engineering Science, Peking University, Beijing 100871, China; pdgxyylb@pku.edu.cn; 2 Yantai Key Laboratory of Characteristic Agricultural Bioresource Conservation & Germplasm Innovative Utilization, School of Life Sciences, Yantai University, Yantai 264006, China; 3 Department of Food Science and Engineering, College of Chemistry and Environmental Engineering, Shenzhen University, Shenzhen 518071, China

**Keywords:** *Chlamydomonas reinhardtii*, mixotrophic cultivation, biomass composition, protein–lipid allocation, light-response modeling, nitrogen starvation

## Abstract

Microalgae are promising platforms for producing biomass and biochemicals, but changes in component concentrations may reflect biomass accumulation, shifts in composition, or both. This study developed a low-dimensional *X*–*ρ*–*A* allocation model for jointly describing and predicting dry biomass (*X*), protein (*P*), and lipid (*L*) dynamics. The model was parameterized and evaluated using mixotrophic Tris–acetate–phosphate (TAP) batch cultures of *Chlamydomonas reinhardtii* strain CC-400. The model uses two composition-derived indices: *ρ*, the fraction of dry biomass represented by the measured protein–lipid module, and *A*, the lipid fraction within this module. Model comparison favored a formulation in which *ρ* was constant over time within each condition. The light-response model was calibrated at 30, 100, and 300 μE m^−2^ s^−1^ and evaluated against an independently generated 500 μE m^−2^ s^−1^ dataset from the same experimental system to assess predictive performance beyond the calibration range. Validation *R*^2^ values were 0.9374, 0.9616, and 0.9321 for dry biomass, protein, and lipid, respectively. Under nitrogen starvation, biomass and protein accumulation were suppressed, whereas *A* increased, indicating lipid enrichment within the measured protein–lipid module. Within this experimental system, the model provides an interpretable framework for predicting light-dependent biomass, protein, and lipid dynamics and distinguishing biomass-driven increases in component concentrations from shifts in protein–lipid allocation.

## 1. Introduction

Microalgae are photosynthetic microbial cell factories whose biotechnological value depends strongly on biomass composition, including proteins, lipids, and carbohydrates [1,2]. Among microalgae, *Chlamydomonas reinhardtii* is a classical model green alga that is commonly cultivated mixotrophically in acetate-containing Tris–acetate–phosphate (TAP) medium [3,4,5]. In *C. reinhardtii*, light and acetate availability affect the abundance and lysine acetylation of proteins involved in photosynthesis, the glyoxylate cycle, and fatty acid metabolism [6]. Light intensity and nitrogen availability can also strongly affect both biomass accumulation and biochemical composition [5,7,8,9,10]. More broadly, the macromolecular composition of *C. reinhardtii* varies with resource availability and growth rate [11]. In this context, protein is a major nitrogen-containing biomass fraction, whereas lipids include product-relevant and storage-associated biomass components [1]. *C. reinhardtii* biomass has also been evaluated as a potential food supplement, with reported essential amino-acid and unsaturated fatty-acid profiles supporting its nutritional relevance [12]. For health-related microalgal bioproducts, such as protein-rich biomass or lipid-containing nutritional ingredients, it is important to determine whether higher protein or lipid concentrations arise mainly from greater biomass accumulation or from enrichment of these components within the biomass [9,10,12,13]. Accordingly, models that jointly describe dry biomass, protein, and lipid dynamics can be useful for interpreting cultivation responses and comparing culture conditions in terms of both production level and biomass composition.

Various kinetic models have been developed to describe microalgal growth and product formation in response to nutrient, substrate, and environmental conditions [14,15,16]. Detailed formulations can further represent intracellular storage pools, substrate uptake, or multiple dynamic state variables [16,17,18]. Recent *C. reinhardtii* models have variously incorporated time-dependent biomass composition, transcriptomic data, quantitative proteomics, and enzyme constraints to represent condition-specific metabolic states [19,20,21]. Depending on their formulation, these approaches can support process evaluation and cultivation optimization or provide mechanistic insight into intracellular metabolism. Their application may, however, require multiple state variables, omics or enzyme-kinetic inputs, and condition-specific calibration [10,15,18,19,21]. When available measurements are limited mainly to biomass, protein, and lipid concentrations, a low-dimensional composition-based model offers a complementary description of their joint dynamics and associated changes in measured biomass composition.

The distinction between concentration and composition is particularly important when evaluating lipid accumulation. Lipid accumulation is commonly quantified using lipid concentration, lipid content, or lipid productivity, but these quantities are not interchangeable [8,9,10,13]. A higher lipid concentration may simply reflect a higher dry biomass concentration, without a corresponding increase in lipid content relative to dry biomass [7,8,9,10,18]. Under nitrogen starvation, growth is suppressed and the protein fraction of dry biomass declines, while starch and lipid fractions change over time [5,8,22]. Recent transcriptomic evidence also supports the time-dependent nature of this response, showing an early nitrogen-scavenging phase followed by later reorganization of carbon metabolism toward storage-compound biosynthesis [23]. These observations highlight the importance of considering biomass composition alongside absolute component concentrations when comparing cultivation responses.

In this study, we aimed to develop a low-dimensional *X*–*ρ*–*A* model to describe and predict dry biomass (*X*), protein (*P*), and lipid (*L*) dynamics. The model was parameterized and evaluated using mixotrophic TAP batch cultures of the cell-wall-deficient *C. reinhardtii* strain CC-400. In this framework, the measured protein–lipid module is defined as the sum of protein and lipid concentrations; *ρ* represents the fraction of dry biomass accounted for by this module, whereas *A* represents the lipid fraction within the module. The light-response model was calibrated with multiple light-intensity datasets and evaluated against an independently generated 500 μE m^−2^ s^−1^ dataset from the same experimental system, while nitrogen starvation was analyzed separately as a contrasting condition for evaluating protein–lipid allocation. Together, the concentration predictions and composition-derived indices enable biomass-driven increases in component concentrations to be distinguished from shifts in protein–lipid allocation under the tested culture conditions.

## 2. Results

To establish the experimental basis for the model, we first examined biomass, protein, and lipid dynamics across different light intensities and under nitrogen starvation. Biomass composition and the derived indices *A* and *ρ* were then used to evaluate whether changes in protein and lipid concentrations reflected biomass-driven accumulation or shifts in composition-derived allocation. Based on these variables, we compared alternative *X–ρ*–*A* formulations and fitted the selected model to the light-response training datasets, while the nitrogen-starvation dataset was analyzed as a condition-specific fit. The resulting light-response model was further evaluated using an independent 500 μE m^−2^ s^−1^ validation dataset.

### 2.1. Light-Dependent Biomass, Protein, and Lipid Accumulation Under TAP Conditions

**Figure 1:**
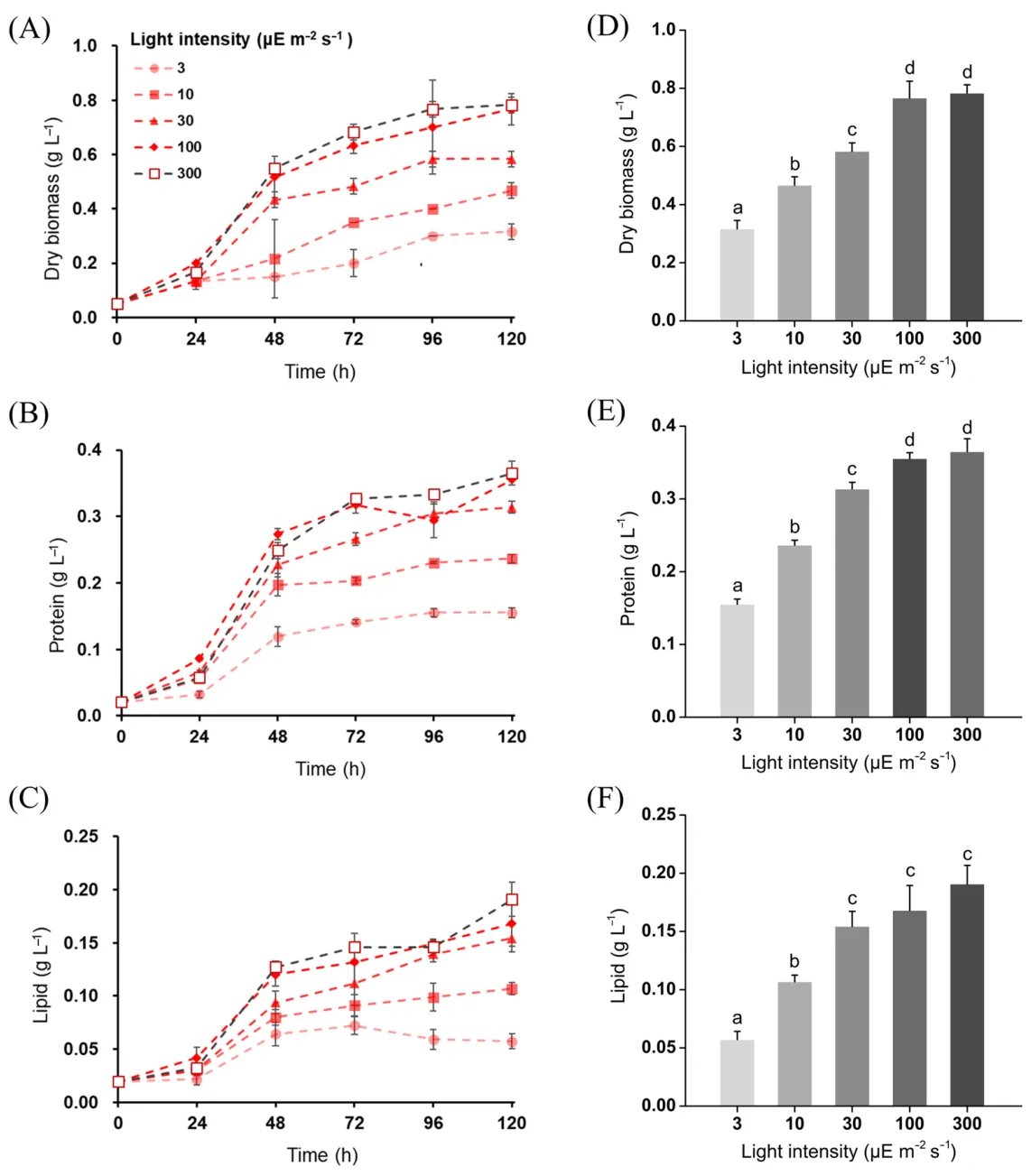
Biomass, protein, and lipid time-course profiles and 120 h endpoint comparisons under different light intensities in Tris–acetate–phosphate (TAP) medium. Temporal changes in dry biomass (**A**), protein (**B**), and lipid (**C**) concentrations of *C. reinhardtii* cultured under 3, 10, 30, 100, and 300 μE m^−2^ s^−1^. Panels (**D**–**F**) show the 120 h endpoint values from the same time-course datasets for statistical comparison among light intensities, including dry biomass (**D**), protein (**E**), and lipid (**F**). Data are presented as mean ± standard deviation (SD) of three biological replicates. Different lowercase letters indicate significant differences among light intensities at 120 h (*p* < 0.05).

Under TAP conditions, dry biomass, protein, and lipid concentrations all increased during cultivation, but their accumulation levels and apparent plateaus differed among light-intensity treatments (Figure 1A–C). All three concentrations increased slowly under the two lowest light intensities, 3 and 10 μE m^−2^ s^−1^, whereas cultures grown under 30–300 μE m^−2^ s^−1^ showed more rapid increases, particularly between 24 and 48 h. After 72–96 h, the concentration curves approached a plateau, with the highest final concentrations generally observed under 100–300 μE m^−2^ s^−1^. Overall, biomass, protein, and lipid concentrations generally increased with irradiance over the lower-to-moderate range, whereas only limited additional accumulation was observed at the upper end of the tested range.

At 120 h, endpoint comparisons further confirmed significant light-dependent differences in dry biomass, protein, and lipid concentrations (Figure 1D–F). Dry biomass increased from approximately 0.32 g L^−1^ at 3 μE m^−2^ s^−1^ to approximately 0.78 g L^−1^ at 300 μE m^−2^ s^−1^, with no significant difference between the 100 and 300 μE m^−2^ s^−1^ treatments. Protein concentration showed a similar pattern, increasing from approximately 0.16 g L^−1^ at 3 μE m^−2^ s^−1^ to approximately 0.36 g L^−1^ at 300 μE m^−2^ s^−1^, and did not differ significantly between the 100 and 300 μE m^−2^ s^−1^ treatments. Lipid concentration increased from approximately 0.06 g L^−1^ at 3 μE m^−2^ s^−1^ to approximately 0.19 g L^−1^ at 300 μE m^−2^ s^−1^, but no significant difference was detected among the 30, 100, and 300 μE m^−2^ s^−1^ treatments at 120 h. Thus, biomass and protein accumulation showed clear plateau-like responses at higher light intensities, whereas lipid concentration showed a higher mean value at 300 μE m^−2^ s^−1^ but greater variability among biological replicates at this light intensity. These light-dependent concentration patterns provided the experimental context for subsequent allocation analysis and modeling.

### 2.2. Nitrogen Starvation Altered Biomass, Protein, and Lipid Accumulation

**Figure 2:**
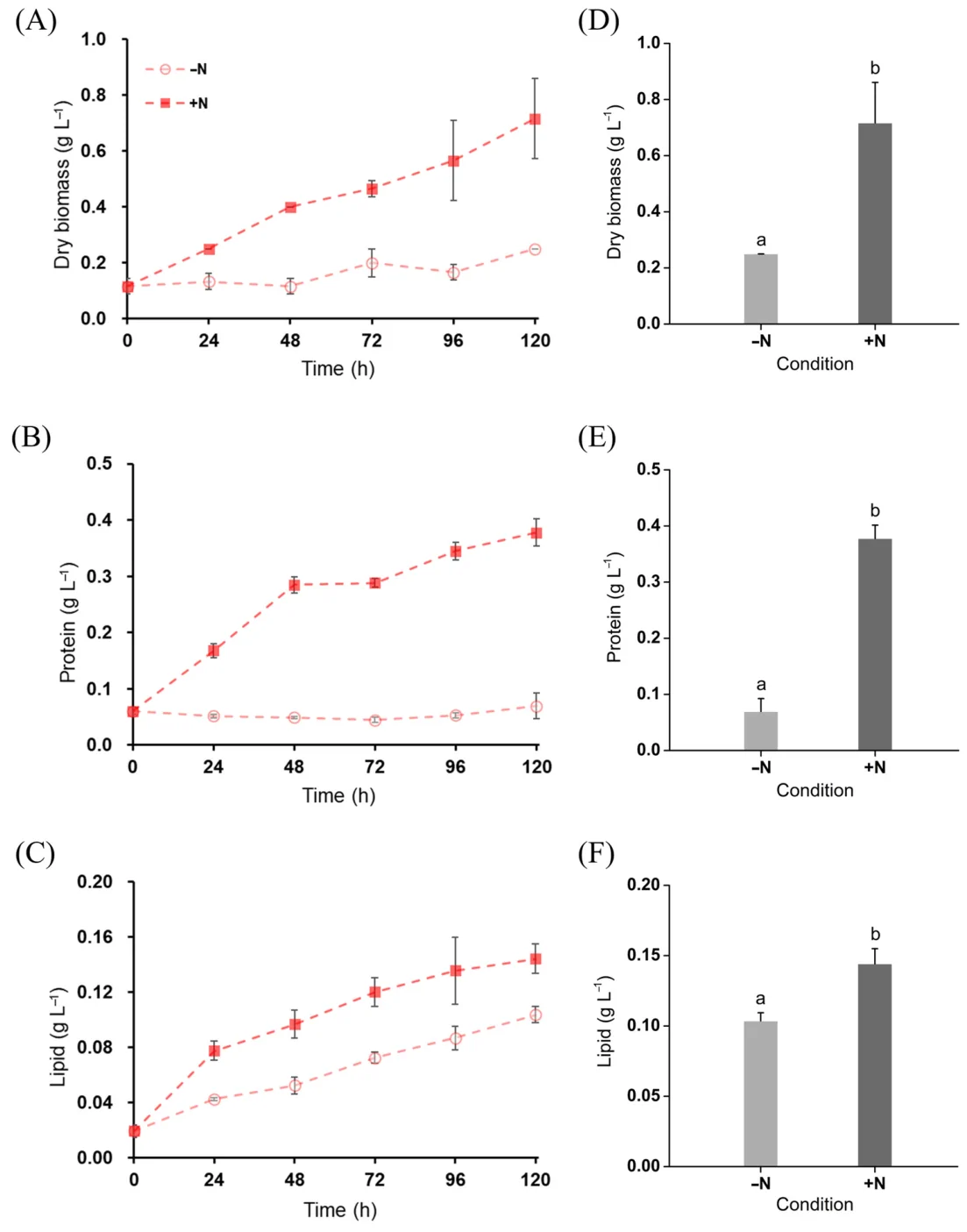
Biomass, protein, and lipid time-course profiles and 120 h endpoint comparisons under TAP and nitrogen-free TAP-N conditions. Temporal changes in dry biomass (**A**), protein (**B**), and lipid (**C**) concentrations of *C. reinhardtii* cultured in TAP medium (+N) or nitrogen-free TAP-N medium (−N) at 30 μE m^−2^ s^−1^. Panels (**D**–**F**) show the 120 h endpoint values from the same time-course datasets for statistical comparison between +N and −N conditions, including dry biomass (**D**), protein (**E**), and lipid (**F**). Data are presented as mean ± SD of three biological replicates. Different lowercase letters indicate significant differences between +N and −N at 120 h (*p* < 0.05).

Nitrogen starvation altered the time-course profiles of biomass, protein, and lipid concentrations under 30 μE m^−2^ s^−1^ compared with the paired nitrogen-replete TAP (+N) control (Figure 2A–C). In TAP medium (+N), dry biomass increased during cultivation, whereas biomass accumulation was strongly suppressed in nitrogen-free TAP medium (TAP-N; −N). The contrast was more pronounced for protein concentration, which increased over time under +N conditions but remained low throughout cultivation under −N conditions. Lipid concentration, however, continued to increase under nitrogen starvation, although its absolute concentration was lower than in the +N control. At 120 h, dry biomass, protein, and lipid concentrations were all significantly lower in −N cultures than in +N cultures (Figure 2D–F), with endpoint values of approximately 0.25 and 0.72 g L^−1^ for dry biomass, 0.07 and 0.38 g L^−1^ for protein, and 0.10 and 0.14 g L^−1^ for lipid in −N and +N cultures, respectively. Thus, the nitrogen-starvation response differed from the light-intensity response, with strongly suppressed biomass and protein accumulation but continued lipid accumulation. This condition therefore provided a contrasting basis for subsequent biomass-composition and allocation analyses.

### 2.3. Biomass Composition and Allocation Indices Showed Distinct Condition-Dependent Patterns

**Figure 3:**
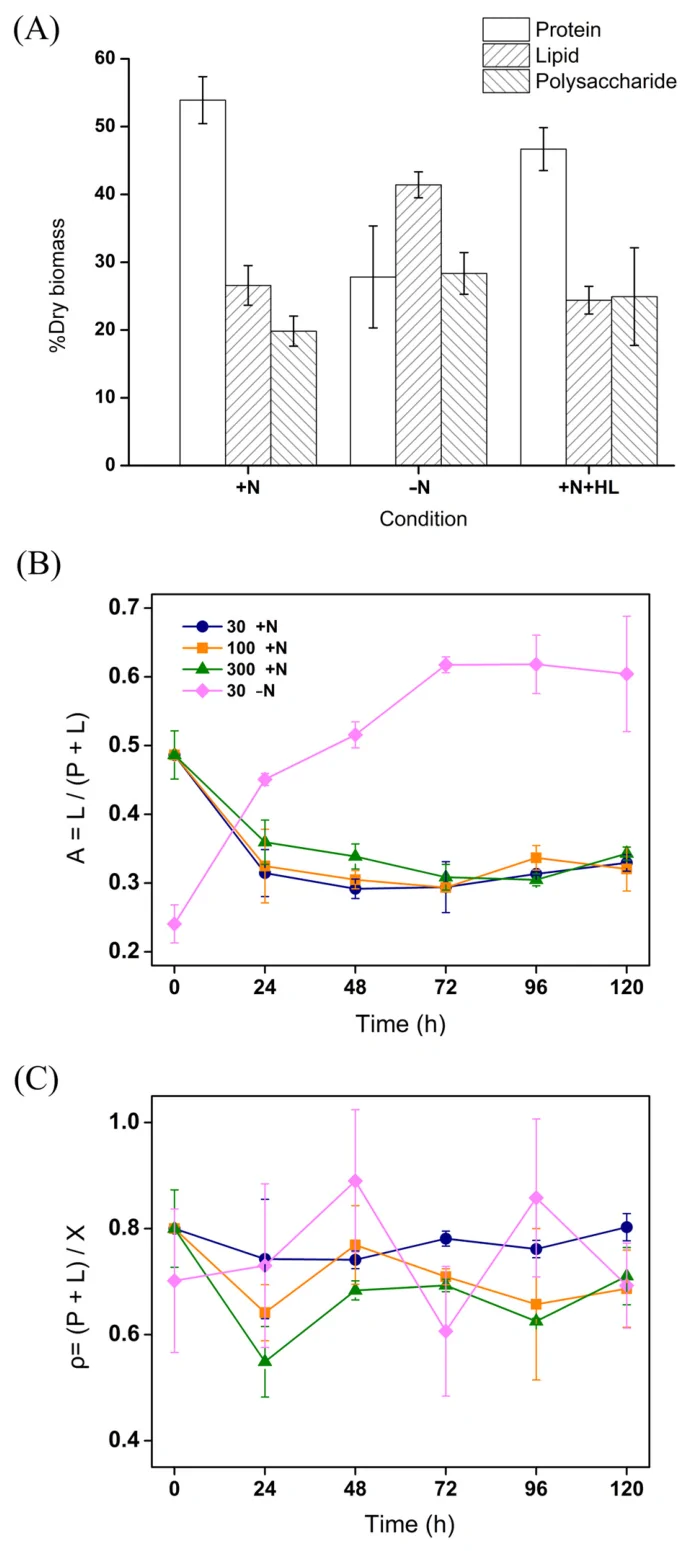
Biomass composition and allocation indices under different light intensities and nitrogen starvation. (**A**) Biomass composition of *C. reinhardtii* at 120 h under representative conditions, including TAP medium at 30 μE m^−2^ s^−1^ (+N), nitrogen-free TAP-N medium at 30 μE m^−2^ s^−1^ (−N), and high-light cultivation in TAP medium at 300 μE m^−2^ s^−1^ (+N+HL). The protein, lipid, and polysaccharide fractions are expressed as percentages of dry biomass. (**B**) Temporal changes in the lipid allocation index *A*, defined as *L*/(*P* + *L*), under 30 +N, 100 +N, 300 +N, and 30 −N conditions. Numbers in these condition labels indicate light intensities in μE m^−2^ s^−1^. *X*, *P*, and *L* denote dry biomass, protein, and lipid concentrations, respectively. (**C**) Temporal changes in the protein–lipid module fraction *ρ*, defined as (*P* + *L*)/*X*, under the same conditions. Data are presented as mean ± SD of three biological replicates. Light intensities are expressed as μE m^−2^ s^−1^.

Biomass-composition analysis further showed differences in the relative fractions of measured biomass components among the tested conditions (Figure 3A). For cultures grown in TAP medium (+N) at 30 μE m^−2^ s^−1^, protein was the largest measured biomass fraction, whereas lipid and polysaccharide accounted for smaller fractions. Under nitrogen starvation, the protein fraction decreased substantially, while the lipid fraction increased and became the largest among the three measured components at 120 h. By contrast, high-light cultivation in TAP medium at 300 μE m^−2^ s^−1^ (+N+HL) showed a protein-dominant composition, although the relative fractions of protein, lipid, and polysaccharide differed from those observed under the 30 μE m^−2^ s^−1^ +N condition. These composition patterns indicate distinct biomass-composition responses under the nitrogen-starvation and high-light conditions examined.

To summarize these composition changes in a reduced modeling framework, two derived indices were calculated from dry biomass *X*, protein *P*, and lipid *L* concentrations: the lipid allocation index *A*, defined as *L*/(*P* + *L*), and the protein–lipid module fraction *ρ*, defined as (*P* + *L*)/*X* (Figure 3B,C). Across the +N light-intensity treatments, the lipid allocation index *A* reached similar values of approximately 0.3–0.4 by the end of cultivation, indicating similar lipid fractions within the protein–lipid module across these light treatments. In contrast, *A* increased under nitrogen starvation and reached a higher late-stage value of approximately 0.60, indicating lipid enrichment within the protein–lipid module. The *ρ* profiles showed a different pattern from *A*: *ρ* showed only minor changes under the 30 μE m^−2^ s^−1^ +N condition, whereas the higher-light and nitrogen-starvation conditions showed lower and/or more variable *ρ* values over time. These results show that *A* and *ρ* captured different aspects of biomass composition. Therefore, both indices were retained for subsequent comparison of alternative *X*–*ρ*–*A* model formulations.

### 2.4. Model Selection Favored the Reduced Constant-ρ X–ρ–A Formulation

**Figure 6:**
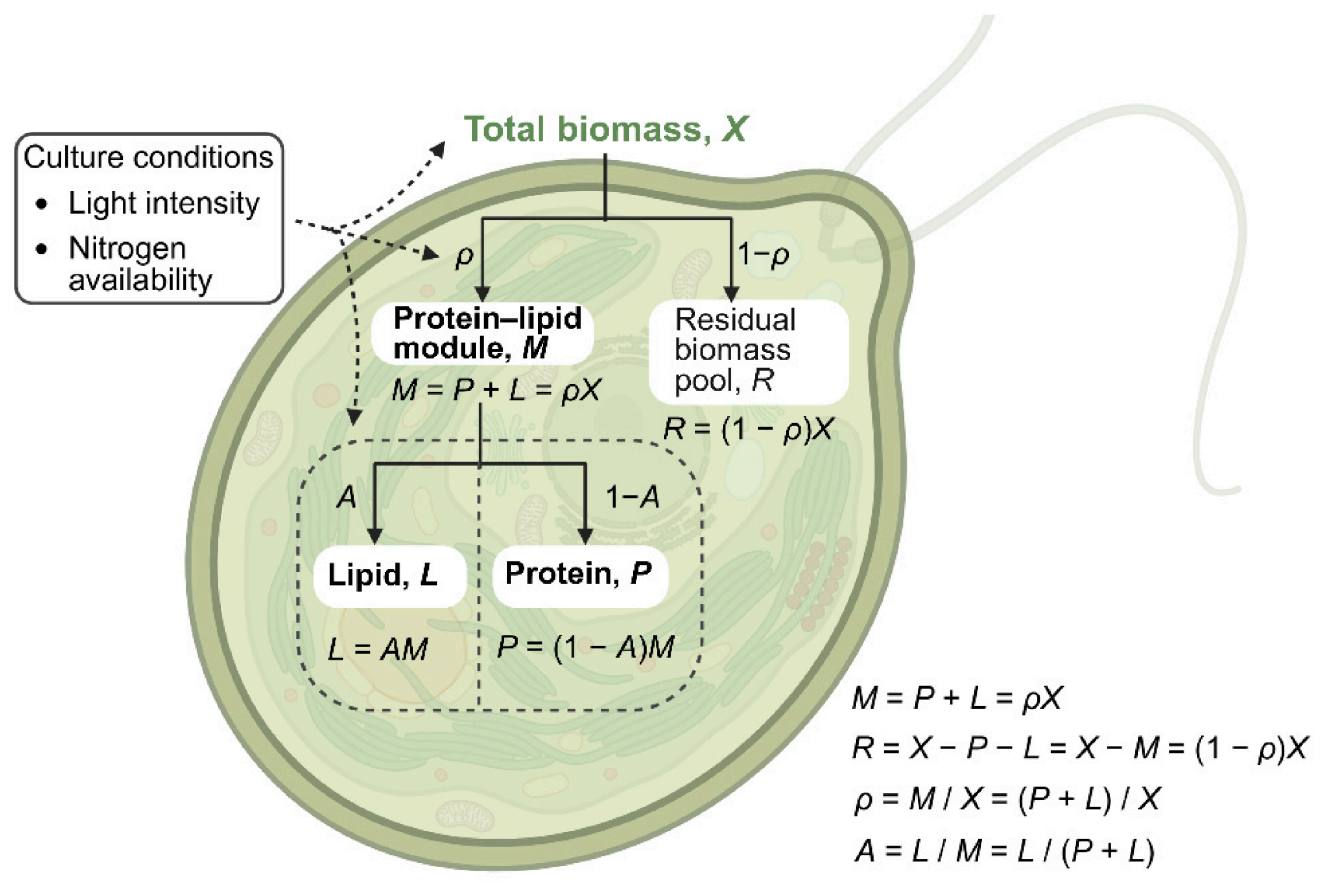
Conceptual framework of the *X*–*ρ*–*A* protein–lipid allocation model. Total biomass *X* is divided into a measured protein–lipid module *M* and a residual biomass pool *R*. The protein–lipid module *M* represents the combined protein and lipid components, whereas *R* represents the remaining biomass pool not included in these two components. The coefficient *ρ* represents the fraction of total biomass represented by the protein–lipid module *M*, whereas 1 − *ρ* represents the residual biomass fraction. Within the protein–lipid module, the lipid allocation index *A* represents the fraction allocated to lipid *L*, whereas 1 − *A* represents the fraction allocated to protein *P*. Dashed arrows indicate culture conditions, including light intensity and nitrogen availability, that may affect *X*, *ρ*, and *A*. These arrows are conceptual inputs rather than fitted transfer terms. For simplicity, condition subscripts *j* and time arguments *t* are omitted in the conceptual diagram.

Following the conceptual framework shown in Figure 6, constant-*ρ* and dynamic-*ρ* variants of the *X*–*ρ*–*A* model were compared to test whether adding a time-dependent term improved the description of the protein–lipid module fraction (Table S1). Across all fitted conditions, including the 30, 100, and 300 μE m^−2^ s^−1^ +N light-response training datasets and the 30 μE m^−2^ s^−1^ −N dataset, the constant-*ρ* variant gave the lowest Akaike information criterion (AIC) and Bayesian information criterion (BIC) values. The dynamic-*ρ* variant included one additional parameter but did not substantially improve model fit, as assessed by root mean squared error (RMSE) and the coefficient of determination (*R*^2^) for dry biomass, protein, and lipid. Thus, the time-dependent relaxation term for *ρ_j_*(*t*) was not supported by the model-selection criteria for the present datasets. The reduced constant-*ρ* formulation was therefore retained as the final model structure for subsequent analyses.

### 2.5. Fitting Performance of the X–ρ–A Model

**Figure 4:**
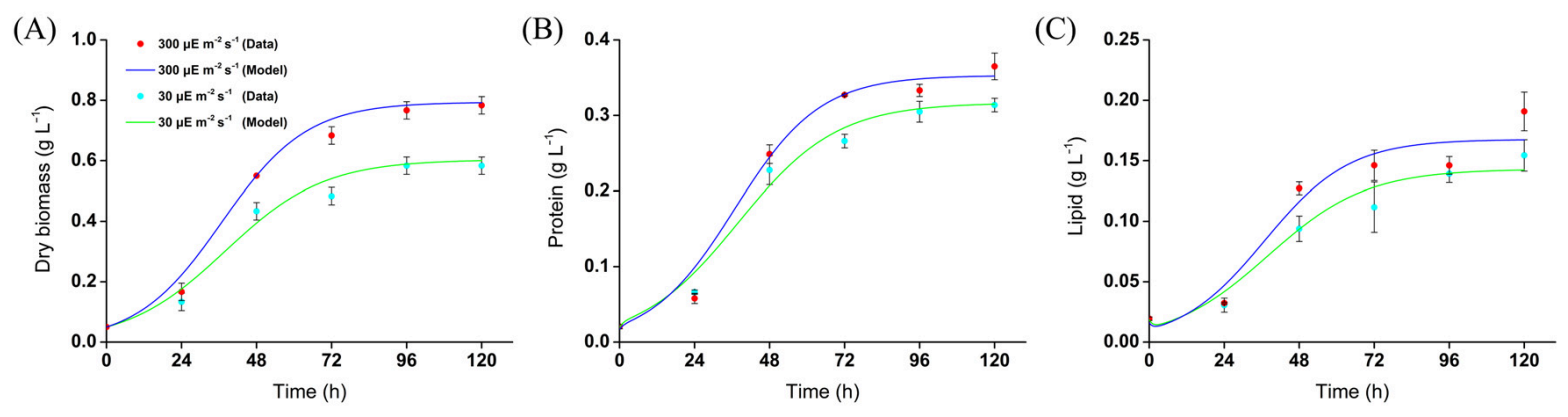
Representative training performance of the *X*–*ρ*–*A* model under light-gradient conditions. Observed and fitted dry biomass (**A**), protein (**B**), and lipid (**C**) concentrations of *C. reinhardtii* at 30 and 300 μE m^−2^ s^−1^ in TAP medium. Symbols represent experimental data, and solid lines represent fitted model curves. Data are presented as mean ± SD of three biological replicates.

Using the selected constant-*ρ* formulation, the *X*–*ρ*–*A* model was fitted to the TAP (+N) light-response training datasets at 30, 100, and 300 μE m^−2^ s^−1^. Representative fits for the 30 and 300 μE m^−2^ s^−1^ conditions showed that the model reproduced the observed time-course trends of dry biomass, protein, and lipid concentrations at both the lower and higher light intensities within the training range (Figure 4). The fitted parameters varied among light intensities, particularly for *K_j_*, *ρ_j_*, and *k_A_*_,*j*_, which summarize condition-specific differences in biomass capacity, protein–lipid module fraction, and the apparent adjustment rate of *A* (Table 1). The condition-specific coefficients of determination were high for all +N light-response training datasets, with *R*^2^*_X_*, *R*^2^*_P_*, and *R*^2^*_L_* ranging from 0.9294 to 0.9880, 0.9606 to 0.9790, and 0.9290 to 0.9487, respectively. These results indicate that the reduced *X*–*ρ*–*A* model jointly described biomass, protein, and lipid concentration dynamics under the +N light-response training conditions.

The selected model structure was also applied to the nitrogen-starvation dataset as a condition-specific descriptive fit for allocation analysis (Figure S1). In nitrogen-free TAP-N medium, the model reproduced the increase in lipid concentration well, whereas the fits for biomass and protein were less accurate than the corresponding fits for the +N training datasets. This contrast was reflected in the condition-specific *R*^2^ values, with a high *R*^2^*_L_* of 0.9664 but lower *R*^2^*_X_* and *R*^2^*_P_* values of 0.5860 and 0.3337, respectively (Table 1). Because biomass accumulation under −N conditions was strongly suppressed and did not approach a clear plateau within the 120 h observation window, the fitted biomass-related parameters should be regarded as weakly constrained and descriptive rather than mechanistic. Therefore, the nitrogen-starvation fit was treated as a descriptive, condition-specific analysis rather than as evidence for a generalized nitrogen-response prediction model. Predictive performance was subsequently evaluated using the independent 500 μE m^−2^ s^−1^ validation dataset.

### 2.6. Independent High-Light Validation of the Light-Response X–ρ–A Model

**Figure 5:**
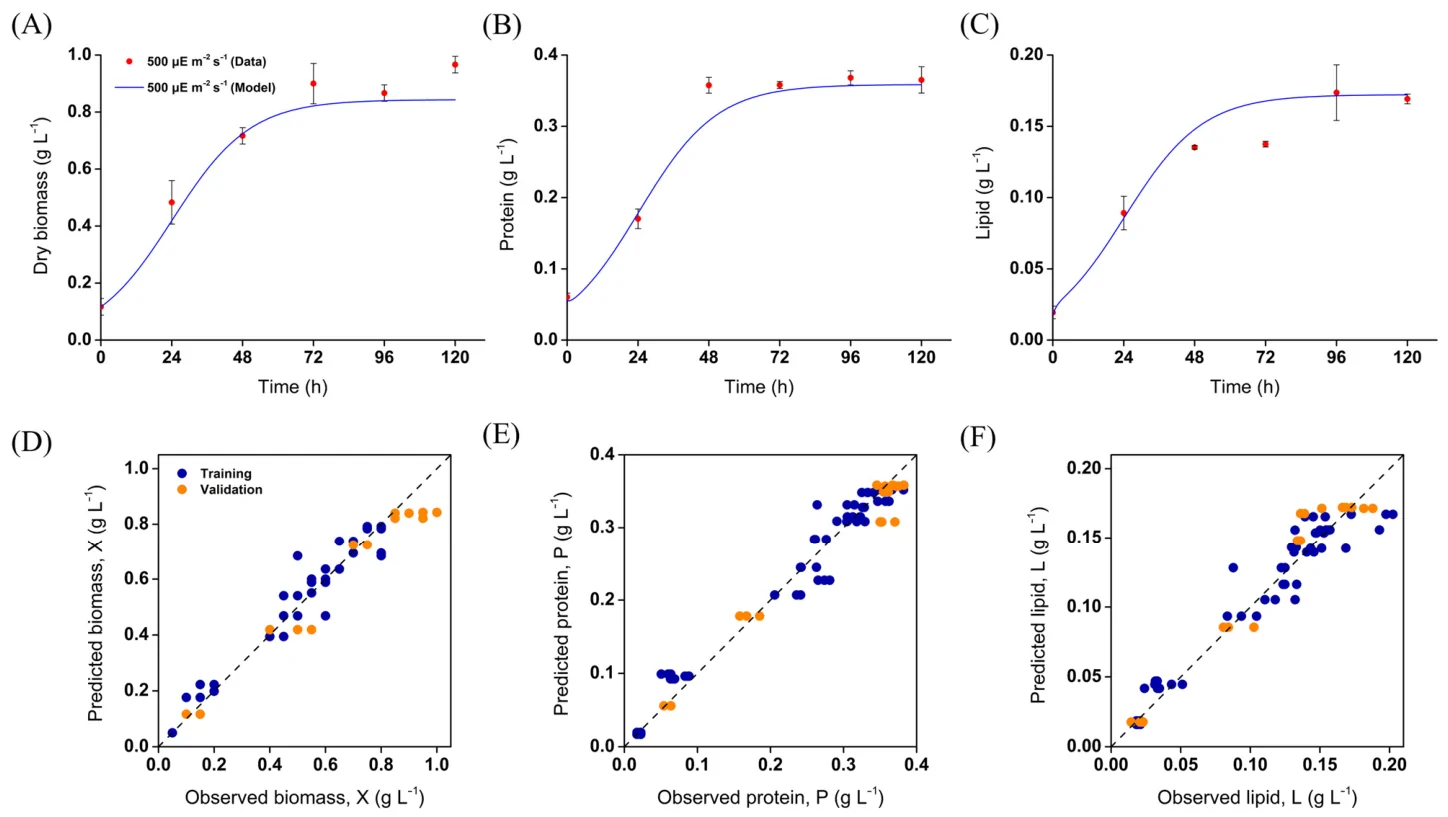
Independent high-light validation and prediction performance of the *X*–*ρ*–*A* model. Observed and predicted dry biomass (**A**), protein (**B**), and lipid (**C**) concentrations of *C. reinhardtii* CC-400 under the independent 500 μE m^−2^ s^−1^ validation condition in TAP medium. Data in panels (**A**–**C**) are presented as mean ± SD of three biological replicates. Symbols represent experimental data, and solid lines represent model predictions. Panels (**D**–**F**) show predicted versus observed values for dry biomass *X* (**D**), protein *P* (**E**), and lipid *L* (**F**). Each point represents one replicate-level observation at a given sampling time. Blue and orange symbols indicate training and validation datasets, respectively, and the dashed line represents the 1:1 identity line. The 500 μE m^−2^ s^−1^ dataset was not used for calibration of the light-response functions; validation predictions were initialized using the measured initial state of the validation culture.

The final light-response *X*–*ρ*–*A* model was evaluated using an independent high-light validation dataset at 500 μE m^−2^ s^−1^. Parameter values for the validation condition, including *μ*(500), *K*(500), *ρ*(500), and *A_∞_*(500), were generated from the light-response functions calibrated using the training datasets at 30, 100, and 300 μE m^−2^ s^−1^, while *k_A_* was assigned the global value *k_A_*_,global_ (Table S2). The 500 μE m^−2^ s^−1^ dataset was not used to calibrate these light-response functions. Predictions were initialized using the measured initial state of the validation culture. Under this condition, the predicted time-course profiles followed the observed increases in dry biomass, protein, and lipid concentrations and their subsequent approach to plateau-like levels at later time points (Figure 5A–C). Minor deviations were observed for the highest late-stage biomass values, with the model slightly underestimating some observations, whereas the protein and lipid predictions remained close to the measured time-course profiles.

Predicted-versus-observed plots using replicate-level data further showed that both training and validation observations lay close to the 1:1 identity line for dry biomass, protein, and lipid (Figure 5D–F). For the independent 500 μE m^−2^ s^−1^ validation dataset, the model achieved *R*^2^ values of 0.9374, 0.9616, and 0.9321 for dry biomass, protein, and lipid, respectively, with corresponding NRMSE values of 11.38%, 8.76%, and 11.98% (Table 2). These values were comparable to the overall training performance, for which *R*^2^ values were 0.9582, 0.9682, and 0.9371 and NRMSE values were 12.00%, 10.43%, and 14.14% for dry biomass, protein, and lipid, respectively. Residual diagnostics indicated that residuals were generally centered near zero across the predicted range, although the highest validation biomass values were slightly underpredicted (Figure S2). Together, these results support the predictive performance of the light-response *X*–*ρ*–*A* model at 500 μE m^−2^ s^−1^ within the same experimental system.

## 3. Discussion

The main contribution of this study is a low-dimensional *X*–*ρ*–*A* model developed and evaluated in TAP batch cultures of *C. reinhardtii* CC-400. The derived variables *ρ* and *A* add composition-level information by describing, respectively, the fraction of dry biomass represented by the measured protein–lipid module and the lipid fraction within that module. Previous kinetic models have used more detailed formulations to describe biomass and product formation. For example, a multi-parametric kinetic model for mixotrophic *C. reinhardtii* incorporated nitrogen and phosphorus quotas, acetate, and average irradiance to predict biomass, starch, and lipid dynamics across nutrient regimes [18], whereas a dynamic model for photoautotrophic *Dunaliella viridis* described light- and nitrogen-regulated carbon partitioning among functional biomass, carbohydrates, and neutral lipids [16]. Both models were further evaluated using experimental conditions that were not used for parameter fitting, providing a test of predictive performance outside the fitting conditions. These formulations provide broader condition coverage or greater process resolution but require time-course measurements of nutrient variables and additional cellular components. By comparison, the *X*–*ρ*–*A* framework is calibrated using routine time-series measurements of dry biomass, total protein, and total lipid, and therefore has lower data requirements than these more detailed formulations.

At 120 h, dry biomass and protein concentrations did not differ significantly between the 100 and 300 μE m^−2^ s^−1^ treatments under TAP conditions. The mean lipid concentration was highest at 300 μE m^−2^ s^−1^, but differences among the 30, 100, and 300 μE m^−2^ s^−1^ treatments were not significant (Figure 1). Together, these results indicate that only limited additional accumulation occurred at the upper end of the tested irradiance range by 120 h. A qualitatively similar irradiance response has been reported for the wall-containing *C. reinhardtii* strain CC-125 in nitrogen-replete TAP cultures, where biomass and protein increased with irradiance and then approached a plateau, whereas the lipid response at high irradiance was comparatively weak [7]. However, unmeasured changes in culture pH or extracellular nutrient availability may also have contributed to the late-stage convergence among the higher-irradiance treatments. Because all cultures in the present study contained acetate, the observed responses to light intensity should also be interpreted in the context of acetate-supported mixotrophic growth. Previous work has shown that light and acetate both influence the proteome and lysine acetylome of *C. reinhardtii* and that acetate-supported mixotrophy is associated with shifts in central carbon fluxes and biomass composition [6,24]. Whether the light-response pattern observed here extends to other light spectra or to photoautotrophic cultures remains to be determined.

The allocation index *A* was used to distinguish changes in lipid concentration from changes in the lipid fraction of the measured protein–lipid module. In the +N treatments at 30, 100, and 300 μE m^−2^ s^−1^, late-stage *A* values were similar (Figure 3), suggesting that the balance between lipid and protein within the measured module changed little across this range of light intensities. Previous studies have also shown that lipid concentration, cellular lipid content, and volumetric lipid productivity do not necessarily change in parallel under different light conditions [13,25,26]. Nitrogen starvation produced a clearer divergence between lipid concentration and *A*: lipid concentration was lower than in the paired +N control, but *A* increased substantially while biomass and protein accumulation were suppressed. Growth limitation accompanied by lipid or storage-compound enrichment under nitrogen deficiency has also been reported in *C. reinhardtii* [5,8,27]. In the wall-containing strain CCAP11/32C under TAP-based mixotrophic cultivation, nitrogen limitation likewise reduced biomass and increased the cellular lipid fraction [28]. Stable-isotope tracing in *C. reinhardtii* has further shown that nitrogen deprivation can involve substantial transfer of fatty acids from membrane lipids to triacylglycerol [29]. In the present study, the contrasting changes in lipid concentration and *A* show why the two measures should be considered together. Although the marked increase in *A* observed under the TAP-N condition was not evident across the TAP light treatments, this contrast does not demonstrate nutrient sufficiency throughout cultivation or exclude partial or late-stage nutrient limitation.

The independently generated 500 μE m^−2^ s^−1^ dataset was used to evaluate model prediction beyond the 30–300 μE m^−2^ s^−1^ calibration range. Because the validation was performed within the same CC-400/TAP batch-culture system, it evaluates predictive performance across irradiance conditions rather than transferability across strains, medium compositions, or cultivation systems. In this context, the 500 μE m^−2^ s^−1^ condition was not intended to represent a typical or optimal light intensity for mixotrophic cultivation. The model yielded *R*^2^ values of 0.9374, 0.9616, and 0.9321 for *X*, *P*, and *L*, respectively, with corresponding NRMSE values of 11.38%, 8.76%, and 11.98%. These *R*^2^ values are in a similar numerical range to the mean *r*^2^ of 0.95 reported for a 35-parameter kinetic model of mixotrophic *C. reinhardtii* that was validated under different nutrient conditions [18], although that *r*^2^ was calculated across the combined fitting and validation datasets rather than from the validation datasets alone. Within this scope, the predicted profiles may support preliminary comparisons of irradiance scenarios and harvest windows, consistent with previous model-based optimization of microalgal lipid production [30].

The present framework was developed for light-response prediction in CC-400/TAP batch cultures under mixotrophic cultivation. Its performance under photoautotrophic or heterotrophic conditions has not yet been evaluated and would need to be tested using data generated under those cultivation modes [19,31]. Published studies have reported quantitative strain-dependent variation in storage-compound accumulation, and cell-wall status has been associated with differences in surface-adsorbed phosphorus and phosphorus partitioning in *C. reinhardtii* [32,33]. At the same time, qualitatively comparable irradiance- and nitrogen-related biomass, protein, and lipid responses have been reported in wall-containing strains [7,28]. Thus, although these major response patterns are not restricted to CC-400, the fitted parameters and quantitative light-response relationships obtained for CC-400 should not be assumed to transfer directly across *C. reinhardtii* strain backgrounds without separate validation. Extension to other eukaryotic microalgae or cyanobacteria would likewise require separate validation, as physiological responses can differ even among green microalgae [31]. The −N dataset represents only the nitrogen-starvation condition tested here and does not define a general nitrogen-response relationship.

Culture pH can change during TAP cultivation and influence the physiology of *C. reinhardtii* [34,35]. In a comparable TAP system without external CO_2_ bubbling, culture pH increased from approximately 7.0 to 8.5 as acetate was assimilated [34]. This published trajectory cannot be used to reconstruct the pH of the present cultures, but it shows that the initial pH should not be assumed to remain constant throughout cultivation. Previous TAP-based studies have also monitored residual acetate and total nitrogen over time, and acetate uptake has been quantified directly in CC-400 [28,36]. These studies show that extracellular nutrient concentrations should not be assumed to remain constant during batch cultivation. In the present study, culture pH and extracellular nutrient concentrations were not monitored during the 120 h cultivation. Consequently, nutrient sufficiency throughout cultivation cannot be demonstrated, and neither the onset of nutrient limitation, if any, nor the identity of any limiting medium component can be determined from the present dataset. These unmeasured variables may have acted as time-dependent confounding variables and may have contributed to the observed *X*, *P*, and *L* trajectories. Any effects of unmeasured pH or nutrient changes on biomass accumulation or protein–lipid composition may be reflected in *X* and the composition-derived *A* and *ρ* values. However, the present data cannot separate these effects from the response to irradiance. Accordingly, the *X*–*ρ*–*A* framework describes the overall trajectories observed under each batch-culture condition rather than the separate contributions of irradiance, culture pH, or extracellular nutrient dynamics. Applying the model to different initial pH values or pH-control regimes, other acetate or ammonium concentrations, medium formulations, or cultivation scales would require additional data and, where appropriate, recalibration or extension.

Repeated sampling progressively reduced the working volume during cultivation, which should be considered when interpreting the observed culture dynamics. Although chlorophyll *a* was not measured, future measurements could provide complementary information on photosynthetic acclimation to light and help relate the observed *X*, *P*, and *L* dynamics to changes in photosynthetic performance. Within this experimental scope, the framework describes and predicts *X*, *P*, and *L* dynamics. Extending the model to incorporate additional physiological variables could support broader physiological interpretation and a wider range of applications but would require corresponding measurements and validation.

## 4. Materials and Methods

### 4.1. Strain and Culture Conditions

The cell-wall-deficient strain *C. reinhardtii* CC-400 (cw15, mt+) was obtained from the Chlamydomonas Resource Center (St. Paul, MN, USA). Cell-wall-deficient *C. reinhardtii* backgrounds can facilitate intracellular protein extraction and lipid-droplet recovery [37,38]. CC-400 has also been used in TAP-based quantitative studies of mixotrophic growth, acetate metabolism, nitrogen deprivation, and protein- and lipid-related responses [22,29,36]. Cells were maintained and cultivated in TAP medium under mixotrophic conditions [39]. The standard TAP formulation was used without strain-specific modification. The TAP medium was adjusted to pH 6.0 during preparation. During the subsequent 120 h cultivation, culture pH was not monitored or controlled, and extracellular nutrient concentrations were not measured over time. No external CO_2_ was supplied, and gas exchange with ambient air occurred through a gas-permeable sealing film. Precultures were grown in 500 mL Erlenmeyer flasks containing 200 mL of TAP medium at 23 °C on an orbital shaker (Minquan, Shanghai, China) at 150 rpm under continuous illumination of 30 μE m^−2^ s^−1^. After 5 days of preculture, the preculture suspensions used for each experimental batch were pooled and thoroughly mixed to prepare a common inoculum. The pooled inoculum was then dispensed to the treatment flasks at a 10% (*v*/*v*) inoculation ratio, and its dry biomass concentration was measured.

All main cultures were carried out in 500 mL Erlenmeyer flasks with a working volume of 200 mL. To investigate the effect of light intensity, cultures were grown under 3, 10, 30, 100, and 300 μE m^−2^ s^−1^. The 300 μE m^−2^ s^−1^ treatment represented the highest light intensity within the light-gradient training experiment. An independent TAP culture was grown at 500 μE m^−2^ s^−1^ for validation of the light-response model. To investigate nitrogen starvation, cells were transferred into nitrogen-free TAP medium (TAP-N), prepared by omitting NH_4_Cl from TAP medium, and cultivated at 30 μE m^−2^ s^−1^. A separate N-replete culture was grown at the same light intensity in the same nitrogen-starvation experimental batch and used as the paired +N control. For each condition, three biological replicate flasks were used. Cultures were sampled every 24 h from 0 to 120 h. At each sampling time, 10 mL of culture was withdrawn from each replicate flask for biomass and biochemical analyses. The withdrawn culture was not replaced, and no additional medium was added during cultivation. The resulting decrease in culture volume was the same across all treatments.

The 30, 100, and 300 μE m^−2^ s^−1^ TAP (+N) datasets were used to calibrate the light-response functions, whereas the 500 μE m^−2^ s^−1^ TAP dataset was reserved for independent validation. The paired 30 μE m^−2^ s^−1^ +N and −N datasets were used for nitrogen-starvation comparison and condition-specific allocation analysis rather than for constructing a generalized nitrogen-response prediction model.

These datasets were generated in three experimental batches: the 3, 10, 30, 100, and 300 μE m^−2^ s^−1^ light-gradient treatments in one batch; the paired 30 μE m^−2^ s^−1^ +N and −N treatments in a second batch; and the 500 μE m^−2^ s^−1^ validation treatment in a third batch. Accordingly, the two 30 μE m^−2^ s^−1^ +N datasets were treated as separate datasets because they were generated in different experimental batches and were not pooled for analysis. The experimental datasets and their roles are summarized in Table 3.

### 4.2. Biomass Determination

Biomass concentration was determined as dry cell weight. Briefly, 2 mL of culture was collected and centrifuged at 5000 rpm for 5 min using an Eppendorf 5424 R centrifuge (Eppendorf SE, Hamburg, Germany). The supernatant was discarded, and the cell pellet was washed twice with deionized water. The washed biomass was collected on pre-weighed dry filter papers and dried in a vacuum oven (Jinghong, Shanghai, China) at 60 °C until constant weight. After cooling to room temperature, the filter papers were weighed again. Biomass concentration was expressed as g L^−1^.

### 4.3. Lipid Determination

Total lipid concentration was determined using the sulfo-phospho-vanillin (SPV) colorimetric method, with minor modifications to the method described by Mishra et al. [40]. A 1 mL aliquot of culture was centrifuged at 5000 rpm for 5 min, and the supernatant was discarded. The wet cell pellet was resuspended in 0.1 mL of ultrapure water. Then, 0.4 mL of concentrated sulfuric acid was added, and the sample was heated at 100 °C for 10 min. The acid–thermal step in the SPV procedure has been reported to degrade pigments and lipophilic proteins [40]. After cooling in an ice bath for 5 min, 1 mL of phospho-vanillin reagent was added. The mixture was incubated at 37 °C for 15 min with shaking at 200 rpm, and the absorbance was measured at 530 nm using a Spark multimode microplate reader (Tecan Group Ltd., Männedorf, Switzerland). Commercial rapeseed oil (Haitian, Foshan, China) was used as the lipid calibration standard. A 1 mg mL^−1^ stock solution was prepared by dissolving 10 mg of rapeseed oil in 10 mL of chloroform. To prepare the calibration standards, different volumes of the stock solution were transferred to tubes, and the chloroform was allowed to evaporate completely. The standards were then subjected to the same SPV procedure as the samples. Lipid concentration was calculated from the resulting calibration curve and expressed as g L^−1^.

### 4.4. Protein Determination

Protein concentration was determined using alkaline extraction followed by a bicinchoninic acid (BCA) assay, based on the method described by Ma et al. [41]. A 1 mL aliquot of culture was centrifuged at 5000 rpm for 5 min, and the supernatant was discarded. The wet biomass pellet was hydrolyzed with 200 μL of 1 M NaOH at 80 °C for 10 min. After hydrolysis, 1.8 mL of water was added, and the sample was centrifuged at 13,000 rpm for 30 min. The supernatant was collected, and total protein concentration was determined using a BCA Protein Assay Kit (Coolaber, Beijing, China; cat. no. SK1070-500T) according to the manufacturer’s instructions. The 5 mg mL^−1^ bovine serum albumin (BSA) standard supplied with the kit was diluted to prepare a series of calibration standards. Protein concentration was calculated from the resulting calibration curve and expressed as g L^−1^.

### 4.5. Polysaccharide Determination

Total polysaccharide concentration was determined as acid-hydrolyzable total sugar using the phenol–sulfuric acid method with minor modifications [41]. A 1 mL aliquot of culture was centrifuged at 5000 rpm for 5 min. The wet biomass pellet was incubated with 200 μL of glacial acetic acid at 80 °C for 10 min. Then, 1.7 mL of acetone was added, followed by centrifugation at 5000 rpm for 10 min. The pellet was resuspended in 0.5 mL of 4 M trifluoroacetic acid and boiled for 4 h. After cooling, the suspension was centrifuged at 13,000 rpm for 5 min. The supernatant was collected for total sugar determination using the phenol–sulfuric acid assay. Polysaccharide concentration was calculated from a glucose standard curve and expressed as glucose equivalents in g L^−1^. Polysaccharide measurements were used only as auxiliary biomass-composition data for endpoint composition analysis and were not included in parameter estimation for the core *X*–*ρ*–*A* model.

### 4.6. Dataset Quality Control

Before model fitting, data quality was checked using predefined criteria. Observations were excluded from parameter estimation if any of the following conditions occurred: missing *X*, *P*, or *L* values; non-positive biomass; negative protein or lipid concentration; *P* + *L* ≤ 0; *P* + *L* ≥ *X*. Excluded values were not used for parameter estimation.

### 4.7. Definition of the X–ρ–A Protein–Lipid Allocation Framework

The conceptual framework of the *X*–*ρ*–*A* model is illustrated in Figure 6. For each experimental condition *j*, biomass, protein, and lipid concentrations at time *t* were denoted as *X_j_*(*t*), *P_j_*(*t*), and *L_j_*(*t*), respectively.

The measured protein–lipid module *M_j_*(*t*) was defined as:

$$M_{j}(t)=P_{j}(t)+L_{j}\left(t\right)$$

The biomass-partition coefficient *ρ_j_*(*t*) was defined as the fraction of total biomass represented by the protein–lipid module:

$$\rho_{j}(t)=\frac{M_{j}\left(t\right)}{X_{j}\left(t\right)}$$

The lipid allocation index *A_j_*(*t*) was defined as the lipid fraction within the protein–lipid module:

$$A_{j}\left(t\right)=\frac{L_{j}\left(t\right)}{M_{j}\left(t\right)}$$

The residual biomass pool *R_j_*(*t*) was calculated as:

$$R_{j}\left(t\right)=X_{j}\left(t\right)-M_{j}\left(t\right)$$

where *R_j_*(*t*) represents the residual biomass concentration associated with components not explicitly modeled in the protein–lipid module, including polysaccharides, pigments, nucleic acids, ash, and other cellular components.

### 4.8. Condition-Specific X–ρ–A Model Formulation

Biomass accumulation was described using a logistic-type function [42]:

$$X_{j}\left(t\right)=\frac{K_{j}}{1+B_{j}e^{-\mu_{j}\left(t-t_{0,j}\right)}}\;$$

where *K_j_* is the condition-specific biomass capacity, *μ_j_* is the apparent biomass growth rate, and *t*_0,*j*_ is the initial sampling time. The coefficient *B_j_* was introduced to simplify notation and was calculated as:

$$B_{j}=\frac{\left(K_{j}-X_{0,j}\right)}{X_{0,j}}$$

where *X*_0,*j*_ is the initial biomass concentration.

The lipid allocation index was described using a first-order relaxation function:

$$A_{j}(t)=A_{\infty ,j}+\left(A_{0,j}-A_{\infty ,j}\right)e^{-k_{A,j}\left(t-t_{0,j}\right)}$$

where *A*_0,*j*_ is the initial lipid allocation index, *A*_∞,*j*_ is the asymptotic lipid allocation index, and *k_A_*_,*j*_ is the apparent rate at which *A_j_*(*t*) approaches *A*_∞,*j*_.

Two alternative formulations were evaluated for *ρ_j_*(*t*). In the full dynamic-*ρ* model, *ρ_j_*(*t*) was allowed to vary over time according to:

$$\rho_{j}(t)=\rho_{\infty ,j}+\left(\rho_{0,j}-\rho_{\infty ,j}\right)e^{-k_{\rho ,j}\left(t-t_{0,j}\right)}$$

where *ρ*_0,*j*_ is the initial protein–lipid module fraction, *ρ*_∞,*j*_ is the asymptotic protein–lipid module fraction, and *k_ρ_*_,*j*_ is the apparent adjustment rate. In the reduced constant-*ρ* model, *ρ_j_*(*t*) was treated as a condition-specific constant:

$$\rho_{j}(t)=\rho_{j}$$

The predicted lipid and protein concentrations were then calculated as:

$$L_{j}\left(t\right)=A_{j}\left(t\right)\rho_{j}X_{j}\left(t\right)$$

$$P_{j}(t)=\left[1-A_{j}\left(t\right)\right]\rho_{j}X_{j}\left(t\right)$$

For the full model with dynamic *ρ_j_*(*t*), *ρ_j_* in the equations above was replaced by *ρ_j_*(*t*). The full and reduced formulations were compared using model-selection criteria, as described below.

### 4.9. Parameter Estimation and Model Selection

Model fitting and numerical analyses were performed in Python 3.12.13. Replicate-level data processing, quality control, and summary statistics were performed using pandas 2.2.2 and NumPy 2.0.2 [43], while nonlinear least-squares parameter estimation was performed using scipy.optimize.least_squares in SciPy 1.16.3 [44]. A robust soft_l1 loss function was used to reduce the influence of occasional observations with large residuals, and parameters were estimated under biologically meaningful bounds. Matplotlib 3.10.0 was used to generate preliminary model diagnostic plots, whereas final publication-quality figures were prepared in Origin Pro 8.5.1. All rate constants were constrained to positive values. *K_j_* was constrained to be greater than the initial biomass *X*_0,*j*_, and *ρ*- and *A*-related parameters were constrained within the interval (0,1).

For each dataset, the initial values *X*_0,*j*_, *ρ*_0,*j*_, and *A*_0,*j*_ were calculated from the first sampling time. The model was fitted to replicate-level *X*, *P*, and *L* data rather than to time point means. Weighted residuals were used to balance the contributions of biomass, protein, and lipid:

$$r=\left[\frac{\left(X_{obs}-X_{pred}\right)}{s_{X}},\frac{\left(P_{obs}-P_{pred}\right)}{s_{P}},\frac{\left(L_{obs}-L_{pred}\right)}{s_{L}}\right]$$

where *s_X_*, *s_P_*, and *s_L_* are variable-specific scaling factors. Each scaling factor was defined as the maximum of the observed standard deviation, 15% of the observed mean, and 10^−3^ g L^−1^.

The full model with dynamic *ρ_j_*(*t*) and the reduced model with constant *ρ_j_* were fitted separately for each dataset. Model variants were compared using the Akaike information criterion (AIC) and Bayesian information criterion (BIC) [45,46]:

$$AIC=nln\left(\frac{{SSE}_{w}}{n}\right)+2k$$

$$BIC=nln\left(\frac{{SSE}_{w}}{n}\right)+kln\left(n\right)$$

where *SSE_w_* is the weighted residual sum of squares, *n* is the number of weighted residuals, and *k* is the number of fitted parameters. Lower AIC and BIC values indicate a better balance between goodness of fit and model complexity. The model variant with the lower AIC was selected as the final condition-specific model, while BIC was used as a supporting model-selection criterion.

### 4.10. Light-Response Parameterization and Independent Validation

To predict biomass, protein, and lipid dynamics under light intensities not used for parameter fitting, a two-stage empirical light-response parameterization was applied. First, condition-specific *X*–*ρ*–*A* parameters were fitted independently for the light-response training datasets at 30, 100, and 300 μE m^−2^ s^−1^. Second, empirical light-response functions were fitted to the condition-specific parameters as functions of light intensity *I*. The functions were fitted on transformed parameter scales to preserve parameter domains: log transformations were used for the positive-valued parameters *μ* and *K*, whereas logit transformations were used for the bounded parameters *ρ* and *A*_∞_. The reference light intensity was set to:

$$I_{ref}=30\;\mu E\;m^{-2}s^{-1}$$

and the transformed light variable was defined as:

$$z_{I}=ln\left(\frac{I}{I_{ref}}\right)$$

The light-response functions were defined as:

$$\ln\left[\mu \left(I\right)\right]=\beta_{\mu ,0}+\beta_{\mu ,1}z_{I}$$

$$ln\left[K\left(I\right)\right]=\beta_{K,0}+\beta_{K,1}z_{I}$$

$$logit\left[\rho \left(I\right)\right]=\beta_{\rho ,0}+\beta_{\rho ,1}z_{I}$$

$$logit\left[A_{\infty }\left(I\right)\right]=\beta_{A,0}+\beta_{A,1}z_{I}$$

where:

$$logit\left(y\right)=ln\left(\frac{y}{1-y}\right)$$

and the inverse-logit transformation was used to obtain *ρ*(*I*) and *A*_∞_(*I*) within the interval (0,1). The global *k_A_* value used for light-response prediction was calculated as the median of the condition-specific *k_A_* values from the light-response training datasets.

The 500 μE m^−2^ s^−1^ dataset was obtained from a separate experimental batch and was not used for fitting the light-response functions. Instead, it was used within the same experimental system to evaluate prediction at an irradiance outside the 30–300 μE m^−2^ s^−1^ calibration range. Given the light intensity and measured initial state of the 500 μE m^−2^ s^−1^ culture, the light-response functions were used to estimate *μ*(*I*), *K*(*I*), *ρ*(*I*), and *A*_∞_(*I*), which were then used to predict *X*(*t*), *P*(*t*), and *L*(*t*). The paired +N and −N datasets were used to evaluate composition-derived allocation responses under nitrogen starvation and were not used for light-response validation.

### 4.11. Model Evaluation and Residual Diagnostics

Model performance was evaluated using root mean squared error (RMSE), mean absolute error (MAE), coefficient of determination (*R*^2^), and normalized RMSE (NRMSE). RMSE and MAE were calculated as:

$$RMSE=\sqrt{\frac{1}{n}\sum_{i=1}^{n}{\left(Y_{obs,i}-Y_{pred,i}\right)}^{2}}$$

$$MAE=\frac{1}{n}\sum_{i=1}^{n}\left|Y_{obs,i}-Y_{pred,i}\right|$$

$$R^{2}=1-\frac{\sum_{i}{\left(Y_{obs,i}-Y_{pred,i}\right)}^{2}}{\sum_{i}{\left(Y_{obs,i}-{\bar{Y}}_{obs}\right)}^{2}}$$

where *Y* represents biomass *X*, protein *P*, or lipid *L*, and ${\bar{Y}}_{obs}$ is the mean observed value of *Y*. NRMSE was calculated by normalizing RMSE to the observed mean:

$$NRMSE=\frac{RMSE}{{\bar{Y}}_{obs}}\times 100$$

Residual diagnostics were performed for the light-response training datasets and the independent 500 μE m^−2^ s^−1^ validation dataset. For each output variable *Y*, residuals were calculated as:

$$e_{Y}=Y_{obs}-Y_{pred}$$

Residuals were plotted against predicted values to assess systematic bias or prediction-dependent error patterns. The N-starvation dataset was used for condition-specific allocation analysis and was therefore not included in the light-response residual diagnostic plot.

### 4.12. Statistical Analysis

Unless otherwise stated, experimental data are presented as mean ± standard deviation (SD) of biological replicates. Normality of the residuals from the endpoint comparisons was assessed using the Shapiro–Wilk test. Endpoint comparisons among multiple light-intensity treatments were performed using one-way analysis of variance (ANOVA) followed by a Tukey test. Comparisons between the +N and −N treatments in the nitrogen-starvation experiment were performed using one-way ANOVA with two groups. Differences were considered statistically significant at *p* < 0.05. Statistical analyses were performed using Origin Pro 8.5.1.

## 5. Conclusions

This study developed a low-dimensional *X*–*ρ*–*A* model to describe dry biomass, protein, and lipid dynamics in mixotrophic TAP batch cultures of *C. reinhardtii* CC-400. The derived indices *A* and *ρ* helped distinguish changes in lipid concentration from changes in the lipid fraction within the measured protein–lipid module and in the fraction of total biomass represented by that module, showing distinct biomass-composition patterns across the tested irradiance treatments and under nitrogen starvation. The selected model used a reduced condition-specific constant-*ρ* formulation while retaining a time-dependent description of lipid allocation through *A*(*t*). The final light-response model adequately described the training datasets and was evaluated using an independently generated 500 μE m^−2^ s^−1^ dataset from the same experimental system, with predictions initialized from the measured initial state of the validation culture. Overall, within this experimental scope, the *X*–*ρ*–*A* model provides a low-dimensional approach for comparing biomass accumulation and protein–lipid allocation across irradiance conditions, while its applicability to other strains, media compositions, or cultivation systems remains to be established.

## Figures and Tables

**Table 1:** Final *X*–*ρ*–*A* model parameters for light-response training and nitrogen-starvation condition-specific fitting. *μ_j_* and *k_A_*_,*j*_ are expressed in h^−1^, *K_j_* is expressed in g L^−1^, and *ρ_j_*, *A_∞_*_,*j*_, and *R*^2^ are dimensionless. *R*^2^*_X_*, *R*^2^*_P_*, and *R*^2^*_L_* indicate condition-specific coefficients of determination for dry biomass *X*, protein *P*, and lipid *L*, respectively.

Dataset ID	Condition	*μ_j_*	*K_j_*	*ρ_j_*	*A_∞_*_,*j*_	*k_A_*_,*j*_	*R* ^2^ *_X_*	*R* ^2^ *_P_*	*R* ^2^ *_L_*
Experiment 3	30 +N	0.0609	0.5939	0.7726	0.3124	0.8442	0.9660	0.9750	0.9378
Experiment 4	100 +N	0.0742	0.7198	0.6828	0.3143	0.9272	0.9294	0.9606	0.9487
Experiment 5	300 +N	0.0702	0.7801	0.6704	0.3233	0.9908	0.9880	0.9790	0.9290
Experiment 7	30 −N	0.0060	5.0000	0.6868	0.6400	0.0305	0.5860	0.3337	0.9664

**Table 2:** Training and independent high-light validation performance of the light-response *X*–*ρ*–*A* model. Training refers to the 30, 100, and 300 μE m^−2^ s^−1^ datasets used to calibrate the light-response functions, whereas validation refers to the independently generated 500 μE m^−2^ s^−1^ dataset from the same experimental system. Metrics were calculated from replicate-level observations. Root mean squared error (RMSE), mean absolute error (MAE), and mean observed values are expressed in g L^−1^, the coefficient of determination (*R*^2^) is dimensionless; and normalized RMSE (NRMSE) is expressed as a percentage of the observed mean.

Dataset Type	Variable	RMSE	MAE	*R* ^2^	Mean Observed	NRMSE (% of Mean)
Training	*X*	0.0542	0.0350	0.9582	0.4519	12.00
Training	*P*	0.0226	0.0165	0.9682	0.2166	10.43
Training	*L*	0.0145	0.0104	0.9371	0.1023	14.14
Validation	*X*	0.0753	0.0577	0.9374	0.6618	11.38
Validation	*P*	0.0241	0.0177	0.9616	0.2753	8.76
Validation	*L*	0.0143	0.0112	0.9321	0.1197	11.98

**Table 3:** Experimental datasets and their roles in light-response modeling and nitrogen-starvation analysis. TAP and nitrogen-free TAP-N medium are denoted as +N and −N, respectively, in subsequent figures and text. The two 30 μE m^−2^ s^−1^ TAP datasets were generated in different batches and were not pooled.

Dataset ID	Light Intensity (μE m^−2^ s^−1^)	Medium	NH_4_Cl in Medium (mM)	Role in Analysis
Experiment 1	3	TAP	7.5	Low-light trend
Experiment 2	10	TAP	7.5	Low-light trend
Experiment 3	30	TAP	7.5	Light-response training
Experiment 4	100	TAP	7.5	Light-response training
Experiment 5	300	TAP	7.5	Light-response training
Experiment 6	500	TAP	7.5	Independent high-light validation
Experiment 7	30	TAP-N	0	N-starvation treatment
Experiment 8	30	TAP	7.5	Paired N-replete control

## Data Availability

The raw data supporting the conclusions of this article will be made available by the authors on request.

## Supplementary Materials

The following supporting information can be downloaded at: https://www.mdpi.com/article/10.3390/molecules31173080/s1. Figure S1. Condition-specific fitting of the *X*–*ρ*–*A* model under nitrogen starvation. Observed and fitted dry biomass (A), protein (B), and lipid (C) concentrations of *Chlamydomonas reinhardtii* cultured in nitrogen-free TAP-N medium (−N) under 30 μE m^−2^ s^−1^. Symbols represent experimental data, and solid lines represent condition-specific model fits. Data are presented as mean ± SD of three biological replicates. The nitrogen-starvation dataset was used for condition-specific fitting and allocation analysis rather than for generalized nitrogen-response validation. Figure S2. Residual diagnostics of the light-response *X*–*ρ*–*A* model. Residuals were plotted against predicted values for dry biomass *X* (A), protein *P* (B), and lipid *L* (C). Residuals were calculated as observed minus predicted values. Each point represents one replicate-level observation. Blue and orange symbols indicate the light-response training datasets and the independent 500 μE m^−2^ s^−1^ validation dataset, respectively. The dashed horizontal line represents zero residual. The nitrogen-starvation dataset was used for condition-specific fitting and allocation analysis and was not included in this residual diagnostic plot. Table S1. Comparison of dynamic *ρ* and constant *ρ* variants of the *X*–*ρ*–*A* model. Table S2. Global light-response functions used for independent high-light validation.

## Abbreviations

**Abbreviation or Notation**	**Definition**	
+N	Nitrogen-replete condition in TAP medium	
−N	Nitrogen-starvation condition in nitrogen-free TAP-N medium	
AIC	Akaike information criterion	
ANOVA	Analysis of variance	
BCA	Bicinchoninic acid	
BIC	Bayesian information criterion	
BSA	Bovine serum albumin	
MAE	Mean absolute error	
NRMSE	Normalized root mean squared error	
RMSE	Root mean squared error	
SD	Standard deviation	
SPV	Sulfo-phospho-vanillin	
TAP	Tris–acetate–phosphate medium	
TAP-N	Nitrogen-free Tris–acetate–phosphate medium	
**Symbol**	**Definition**	**Unit**
*j*	Index of the experimental condition or dataset	—
*t*	Cultivation time	h
*t*_0,*j*_	Initial sampling time	h
*X_j_*(*t*)	Dry biomass concentration	g L^−1^
*P_j_*(*t*)	Protein concentration	g L^−1^
*L_j_*(*t*)	Lipid concentration	g L^−1^
*M_j_*(*t*)	Measured protein–lipid module concentration (sum of protein and lipid concentrations)	g L^−1^
*R_j_*(*t*)	Residual biomass concentration	g L^−1^
*A_j_*(*t*)	Lipid fraction within the measured protein–lipid module	Dimensionless
*ρ_j_*(*t*)	Time-dependent protein–lipid module fraction	Dimensionless
*ρ_j_*	Condition-specific constant protein–lipid module fraction	Dimensionless
*μ_j_*	Apparent biomass growth-rate parameter	h^−1^
*K_j_*	Biomass-capacity parameter	g L^−1^
*X*_0,*j*_	Initial dry biomass concentration	g L^−1^
*A*_0,*j*_	Initial lipid allocation index	Dimensionless
*A*_∞__,*j*_	Asymptotic lipid allocation index	Dimensionless
*k_A_*_,*j*_	Apparent adjustment rate of *A_j_*(*t*) toward *A*_∞,*j*_	h^−1^
*ρ*_0,*j*_	Initial protein–lipid module fraction	Dimensionless
*ρ*_∞__,*j*_	Asymptotic protein–lipid module fraction	Dimensionless
*k_ρ_*_,*j*_	Apparent adjustment rate of *ρ_j_*(*t*) toward *ρ*_∞,*j*_	h^−1^
*I*	Light intensity	μE m^−2^ s^−1^
*z_I_*	Log-transformed relative light intensity, *z_I_* = ln(*I*/*I*_ref_), where *I*_ref_ is the reference light intensity	Dimensionless
*R* ^2^	Coefficient of determination	Dimensionless
*R* ^2^ *_X_*	Coefficient of determination for dry biomass concentration	Dimensionless
*R* ^2^ *_P_*	Coefficient of determination for protein concentration	Dimensionless
*R* ^2^ *_L_*	Coefficient of determination for lipid concentration	Dimensionless

## References

[1] Udayan A., Pandey A.K., Sirohi R., Sreekumar N., Sang B.-I., Sim S.J., Kim S.H., Pandey A. (2023). Production of Microalgae with High Lipid Content and Their Potential as Sources of Nutraceuticals. Phytochem. Rev..

[2] Olguín E.J., Sánchez-Galván G., Arias-Olguín I.I., Melo F.J., González-Portela R.E., Cruz L., De Philippis R., Adessi A. (2022). Microalgae-Based Biorefineries: Challenges and Future Trends to Produce Carbohydrate Enriched Biomass, High-Added Value Products and Bioactive Compounds. Biology.

[3] Salomé P.A., Merchant S.S. (2019). A Series of Fortunate Events: Introducing *Chlamydomonas* as a Reference Organism. Plant Cell.

[4] Pessoa J.d.S., de Oliveira C.F.M., Mena-Chalco J.P., de Carvalho J.C.M., Ferreira-Camargo L.S. (2023). Trends on *Chlamydomonas reinhardtii* Growth Regimes and Bioproducts. Biotechnol. Appl. Biochem..

[5] Yang L., Chen J., Qin S., Zeng M., Jiang Y., Hu L., Xiao P., Hao W., Hu Z., Lei A. (2018). Growth and Lipid Accumulation by Different Nutrients in the Microalga *Chlamydomonas reinhardtii*. Biotechnol. Biofuels.

[6] Füßl M., König A.-C., Eirich J., Hartl M., Kleinknecht L., Bohne A.-V., Harzen A., Kramer K., Leister D., Nickelsen J. (2022). Dynamic Light- and Acetate-Dependent Regulation of the Proteome and Lysine Acetylome of *Chlamydomonas*. Plant J..

[7] Li X., Huff J., Crunkleton D.W., Johannes T.W. (2023). Light Intensity and Spectral Quality Modulation for Improved Growth Kinetics and Biochemical Composition of *Chlamydomonas reinhardtii*. J. Biotechnol..

[8] Kamalanathan M., Pierangelini M., Shearman L.A., Gleadow R., Beardall J. (2016). Impacts of Nitrogen and Phosphorus Starvation on the Physiology of *Chlamydomonas reinhardtii*. J. Appl. Phycol..

[9] Zheng S., Zou S., Wang H., Feng T., Sun S., Chen H., Wang Q. (2022). Reducing Culture Medium Nitrogen Supply Coupled with Replenishing Carbon Nutrient Simultaneously Enhances the Biomass and Lipid Production of *Chlamydomonas reinhardtii*. Front. Microbiol..

[10] Figueroa-Torres G.M., Pittman J.K., Theodoropoulos C. (2022). A Highly Productive Mixotrophic Fed-Batch Strategy for Enhanced Microalgal Cultivation. Sustain. Energy Fuels.

[11] Isanta-Navarro J., Peoples L.M., Bras B., Church M.J., Elser J.J. (2024). Elemental and Macromolecular Plasticity of *Chlamydomonas reinhardtii* (Chlorophyta) in Response to Resource Limitation and Growth Rate. J. Phycol..

[12] Darwish R., Gedi M.A., Akepach P., Assaye H., Zaky A.S., Gray D.A. (2020). *Chlamydomonas reinhardtii* Is a Potential Food Supplement with the Capacity to Outperform *Chlorella* and *Spirulina*. Appl. Sci..

[13] Shokravi Z., Shokravi H., Chyuan O.H., Lau W.J., Koloor S.S., Petrů M., Ismail A.F. (2020). Improving ‘Lipid Productivity’ in Microalgae by Bilateral Enhancement of Biomass and Lipid Contents: A Review. Sustainability.

[14] Lee E., Jalalizadeh M., Zhang Q. (2015). Growth Kinetic Models for Microalgae Cultivation: A Review. Algal Res..

[15] Bekirogullari M., Figueroa-Torres G.M., Pittman J.K., Theodoropoulos C. (2020). Models of Microalgal Cultivation for Added-Value Products—A Review. Biotechnol. Adv..

[16] Wang D., Lai Y.-C., Karam A.L., de los Reyes F.L., Ducoste J.J. (2019). Dynamic Modeling of Microalgae Growth and Lipid Production under Transient Light and Nitrogen Conditions. Environ. Sci. Technol..

[17] Bekirogullari M., Pittman J.K., Theodoropoulos C. (2018). Multi-Factor Kinetic Modelling of Microalgal Biomass Cultivation for Optimised Lipid Production. Bioresour. Technol..

[18] Figueroa-Torres G.M., Pittman J.K., Theodoropoulos C. (2021). Optimisation of Microalgal Cultivation via Nutrient-Enhanced Strategies: The Biorefinery Paradigm. Biotechnol. Biofuels.

[19] Yao H., Dahal S., Yang L. (2023). Novel Context-Specific Genome-Scale Modelling Explores the Potential of Triacylglycerol Production by *Chlamydomonas reinhardtii*. Microb. Cell Factories.

[20] Metcalf A.J., Boyle N.R. (2022). Rhythm of the Night (and Day): Predictive Metabolic Modeling of Diurnal Growth in *Chlamydomonas*. mSystems.

[21] Arend M., Zimmer D., Xu R., Sommer F., Mühlhaus T., Nikoloski Z. (2023). Proteomics and Constraint-Based Modelling Reveal Enzyme Kinetic Properties of *Chlamydomonas reinhardtii* on a Genome Scale. Nat. Commun..

[22] Park J.-J., Wang H., Gargouri M., Deshpande R.R., Skepper J.N., Holguin F.O., Juergens M.T., Shachar-Hill Y., Hicks L.M., Gang D.R. (2015). The Response of *Chlamydomonas reinhardtii* to Nitrogen Deprivation: A Systems Biology Analysis. Plant J..

[23] Lee J.-H., Munz J., Dharmasiri H.N., Jin E., Joo S. (2025). Dissecting Nitrogen Starvation Signaling in *Chlamydomonas*: Insights from Arginine-Fed Transcriptome Profiling. Algal Res..

[24] Koley S., Foley K., Perrine Z., Morley S.A., Kambhampati S., Gomez O., Chu K.L., Chou Y.-H., Wei M., Tzeng S.-C. (2026). Metabolic Rewiring and Biomass Redistribution Enable Optimized Mixotrophic Growth in *Chlamydomonas*. Proc. Natl. Acad. Sci. USA.

[25] Seo S.-H., Ha J.-S., Yoo C., Srivastava A., Ahn C.-Y., Cho D.-H., La H.-J., Han M.-S., Oh H.-M. (2017). Light Intensity as Major Factor to Maximize Biomass and Lipid Productivity of *Ettlia* sp. in CO_2_-Controlled Photoautotrophic Chemostat. Bioresour. Technol..

[26] Abdelkarim O.H., Wijffels R.H., Barbosa M.J. (2025). Microalgal Lipid Production: A Comparative Analysis of *Nannochloropsis* and *Microchloropsis* Strains. J. Appl. Phycol..

[27] Dao O., Burlacot A., Buchert F., Bertrand M., Auroy P., Stoffel C., Madireddi S.K., Irby J., Hippler M., Peltier G. (2025). Cyclic and Pseudo-Cyclic Electron Pathways Play Antagonistic Roles during Nitrogen Deficiency in *Chlamydomonas reinhardtii*. Plant Physiol..

[28] Figueroa-Torres G.M., Pittman J.K., Theodoropoulos C. (2017). Kinetic Modelling of Starch and Lipid Formation during Mixotrophic, Nutrient-Limited Microalgal Growth. Bioresour. Technol..

[29] Young D.Y., Shachar-Hill Y. (2021). Large Fluxes of Fatty Acids from Membranes to Triacylglycerol and Back during N-Deprivation and Recovery in *Chlamydomonas*. Plant Physiol..

[30] Bekirogullari M., Fragkopoulos I.S., Pittman J.K., Theodoropoulos C. (2017). Production of Lipid-Based Fuels and Chemicals from Microalgae: An Integrated Experimental and Model-Based Optimization Study. Algal Res..

[31] Young E.B., Reed L., Berges J.A. (2022). Growth Parameters and Responses of Green Algae across a Gradient of Phototrophic, Mixotrophic and Heterotrophic Conditions. PeerJ.

[32] Siaut M., Cuiné S., Cagnon C., Fessler B., Nguyen M., Carrier P., Beyly A., Beisson F., Triantaphylidès C., Li-Beisson Y. (2011). Oil Accumulation in the Model Green Alga *Chlamydomonas reinhardtii*: Characterization, Variability between Common Laboratory Strains and Relationship with Starch Reserves. BMC Biotechnol..

[33] Yuan S., Yang Y., Wang X., Shao S., Jia X., Zhu Y., Luo B., Yu J. (2026). Surface-Adsorbed Phosphorus Buffers Environmental Phosphate Availability and Influences Phosphorus Uptake in *Chlamydomonas reinhardtii*. Environ. Technol. Innov..

[34] Hui C., Schmollinger S., Strenkert D., Holbrook K., Montgomery H.R., Chen S., Nelson H.M., Weber P.K., Merchant S.S. (2022). Simple Steps to Enable Reproducibility: Culture Conditions Affecting *Chlamydomonas* Growth and Elemental Composition. Plant J..

[35] Ochoa-Alfaro A.E., Gaytán-Luna D.E., González-Ortega O., Zavala-Arias K.G., Paz-Maldonado L.M.T., Rocha-Uribe A., Soria-Guerra R.E. (2019). pH Effects on the Lipid and Fatty Acids Accumulation in *Chlamydomonas reinhardtii*. Biotechnol. Prog..

[36] Boyle N.R., Morgan J.A. (2009). Flux Balance Analysis of Primary Metabolism in *Chlamydomonas reinhardtii*. BMC Syst. Biol..

[37] Öztürk E., Mittelmeier T., Dieckmann C., Sukatar A. (2026). Optimized Immunoprecipitation Identifies Candidate Proteins Associated with Eyespot Protein EYE2 in *Chlamydomonas reinhardtii*. Turk. J. Bot..

[38] Baumgartner J., Chua S.T., Blunier M., Gao F., Arita-Merino N., Abiusi F., Archer L., Cybulski K., Smith A.G., Studer M.H.-P. (2025). Comparison of Lipid Droplet Extraction from Cell Wall-Deficient *Chlamydomonas reinhardtii* with Pulsed Electric Fields or Osmotic Shock. Bioresour. Technol..

[39] Harris E.H., Harris E.H., Stern D.B., Witman G.B. (2009). *Chlamydomonas* in the Laboratory. The Chlamydomonas Sourcebook.

[40] Mishra S.K., Suh W.I., Farooq W., Moon M., Shrivastav A., Park M.S., Yang J.-W. (2014). Rapid Quantification of Microalgal Lipids in Aqueous Medium by a Simple Colorimetric Method. Bioresour. Technol..

[41] Ma X., Liu J., Liu B., Chen T., Yang B., Chen F. (2016). Physiological and Biochemical Changes Reveal Stress-Associated Photosynthetic Carbon Partitioning into Triacylglycerol in the Oleaginous Marine Alga *Nannochloropsis oculata*. Algal Res..

[42] Zwietering M.H., Jongenburger I., Rombouts F.M., van ’t Riet K. (1990). Modeling of the Bacterial Growth Curve. Appl. Environ. Microbiol..

[43] Harris C.R., Millman K.J., van der Walt S.J., Gommers R., Virtanen P., Cournapeau D., Wieser E., Taylor J., Berg S., Smith N.J. (2020). Array Programming with NumPy. Nature.

[44] Virtanen P., Gommers R., Oliphant T.E., Haberland M., Reddy T., Cournapeau D., Burovski E., Peterson P., Weckesser W., Bright J. (2020). SciPy 1.0: Fundamental Algorithms for Scientific Computing in Python. Nat. Methods.

[45] Schwarz G. (1978). Estimating the Dimension of a Model. Ann. Stat..

[46] Akaike H. (1974). A New Look at the Statistical Model Identification. IEEE Trans. Autom. Control.

