# Supplementary material for: A Low-Dimensional X–ρ–A Allocation Model for Predicting Light-Dependent Biomass, Protein, and Lipid Dynamics in TAP Batch Cultures of Chlamydomonas reinhardtii CC-400
Source: Molecules. 2026 Sep 1;31(17):3080. doi: 10.3390/molecules31173080 (PMC13567679; doi:10.3390/molecules31173080)
Supplement: Supplementary file 1 [file molecules-31-03080-s001.zip › molecules-4457637-supplementary.pdf]

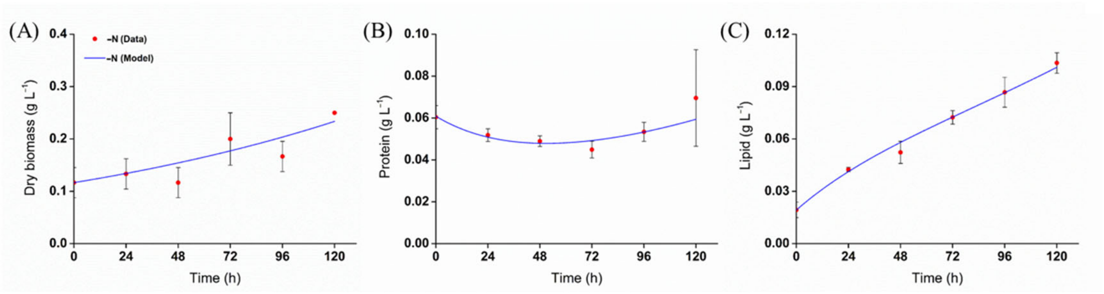

Figure S1. Condition-specific fitting of the *X*–*ρ*–*A* model under nitrogen starvation. Observed and fitted dry biomass (A), protein (B), and lipid (C) concentrations of *Chlamydomonas reinhardtii* cultured in nitrogen-free TAP-N medium (−N) under 30 μE m^{-2} s^{-1}. Symbols represent experimental data, and solid lines represent condition-specific model fits. Data are presented as mean ± SD of three biological replicates. The nitrogen-starvation dataset was used for condition-specific fitting and allocation analysis rather than for generalized nitrogen-response validation.

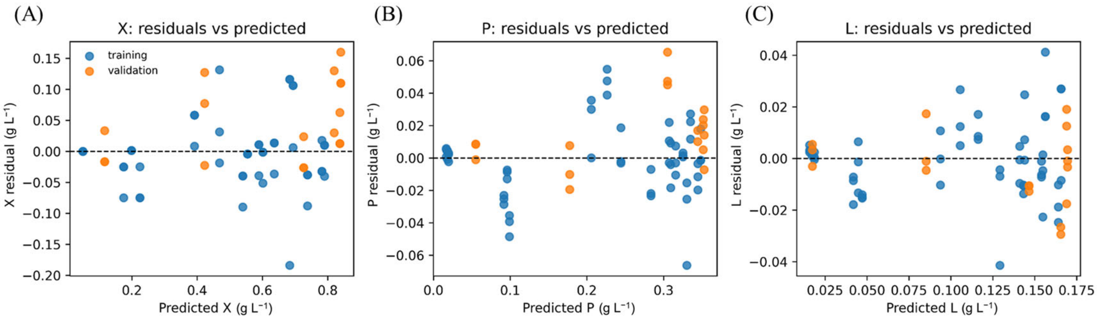

Figure S2. Residual diagnostics of the light-response *X–ρ–A* model. Residuals were plotted against predicted values for dry biomass *X* (A), protein *P* (B), and lipid *L* (C). Residuals were calculated as observed minus predicted values. Each point represents one replicate-level observation. Blue and orange symbols indicate the light-response training datasets and the independent 500 μE m^{-2} s^{-1} validation dataset, respectively. The dashed horizontal line represents zero residual. The nitrogen-starvation dataset was used for condition-specific fitting and allocation analysis and was not included in this residual diagnostic plot.

Table S1. Comparison of dynamic *ρ* and constant *ρ* variants of the *X*–*ρ*–*A* model.

Light intensity (μE m^{-2} s^{-1})	Nitrogen	Model variant	No. of parameters	AIC	ΔAIC	BIC	ΔBIC	$RMSE_X$	$RMSE_P$	$RMSE_L$	R^2_X	R^2_P	R^2_L	Selected
30	+N	constant ρ	5	-163.2823	0.0000	-153.3373	0.0000	0.0390	0.0182	0.0129	0.9660	0.9750	0.9378	√
30	+N	dynamic ρ	6	-161.2962	1.9860	-149.3623	3.9750	0.0390	0.0182	0.0129	0.9660	0.9750	0.9378	
100	+N	constant ρ	5	-147.8646	0.0000	-137.9197	0.0000	0.0724	0.0249	0.0126	0.9294	0.9606	0.9487	√
100	+N	dynamic ρ	6	-146.0305	1.8341	-134.0966	3.8231	0.0724	0.0249	0.0126	0.9293	0.9608	0.9492	
300	+N	constant ρ	5	-171.5850	0.0000	-161.6401	0.0000	0.0317	0.0198	0.0169	0.9880	0.9790	0.9290	√
300	+N	dynamic ρ	6	-169.8458	1.7392	-157.9119	3.7282	0.0317	0.0198	0.0168	0.9880	0.9792	0.9294	
30	-N	constant ρ	5	-43.4996	0.0000	-33.5547	0.0000	0.0353	0.0094	0.0052	0.5860	0.3337	0.9664	√
30	-N	dynamic ρ	6	-41.3646	2.1351	-29.4307	4.1240	0.0353	0.0094	0.0052	0.5858	0.3314	0.9661	

Note: +N and −N denote standard TAP and nitrogen-free TAP media, respectively. The constant *ρ* variant assumes a time-invariant ρ_j within each condition.

Table S2. Global light-response functions used for independent high-light validation.

Parameter function	Transform	Equation	Intercept β_0	Slope β_1	Interpretation
$\mu(I)$	log	$\ln[\mu(I)]=\beta_0+\beta_1 z_I$	-2.7598	0.0635	light-dependent growth rate
$K(I)$	log	$\ln[K(I)]=\beta_0+\beta_1 z_I$	-0.5052	0.1191	light-dependent biomass carrying capacity
ρ(I)	logit	$\text{logit}[\rho(I)]=\beta_0+\beta_1 z_I$	1.1633	-0.2253	light-dependent protein–lipid module fraction
$A_\infty(I)$	logit	$\text{logit}[A_\infty(I)]=\beta_0+\beta_1 z_I$	-0.7945	0.0216	light-dependent lipid allocation asymptote
$k_{A,\text{global}}$	constant	$k_{A,\text{global}}=\text{median}(k_{\text{A,j}})$	0.9272*	—	Global transition-rate constant for A(t)

*For $k_{A,\text{global}}$, the value shown under the intercept column represents the global constant estimate rather than a transformed-scale intercept.
